# The Multifaceted Activity of the VirF Regulatory Protein in the *Shigella* Lifestyle

**DOI:** 10.3389/fmolb.2016.00061

**Published:** 2016-09-29

**Authors:** Maria Letizia Di Martino, Maurizio Falconi, Gioacchino Micheli, Bianca Colonna, Gianni Prosseda

**Affiliations:** ^1^Dipartimento di Biologia e Biotecnologie C. Darwin, Istituto Pasteur Italia-Fondazione Cenci Bolognetti, Sapienza Università di RomaRoma, Italy; ^2^Laboratorio di Genetica Molecolare e dei Microrganismi, Scuola di Bioscienze e Medicina Veterinaria, Università di CamerinoCamerino, Italy; ^3^Istituto di Biologia e Patologia Molecolari, Consilglio Nazionale Delle RichercheRoma, Italy

**Keywords:** VirF, *Shigella*, pathogenic *E. coli*, AraC proteins, transcriptional regulators, DNA binding proteins, bacterial virulence, antivirulence therapy

## Abstract

*Shigella* is a highly adapted human pathogen, mainly found in the developing world and causing a severe enteric syndrome. The highly sophisticated infectious strategy of *Shigella* banks on the capacity to invade the intestinal epithelial barrier and cause its inflammatory destruction. The cellular pathogenesis and clinical presentation of shigellosis are the sum of the complex action of a large number of bacterial virulence factors mainly located on a large virulence plasmid (pINV). The expression of pINV genes is controlled by multiple environmental stimuli through a regulatory cascade involving proteins and sRNAs encoded by both the pINV and the chromosome. The primary regulator of the virulence phenotype is VirF, a DNA-binding protein belonging to the AraC family of transcriptional regulators. The *virF* gene, located on the pINV, is expressed only within the host, mainly in response to the temperature transition occurring when the bacterium transits from the outer environment to the intestinal milieu. VirF then acts as anti-H-NS protein and directly activates the *icsA* and *virB* genes, triggering the full expression of the invasion program of *Shigella*. In this review we will focus on the structure of VirF, on its sophisticated regulation, and on its role as major player in the path leading from the non-invasive to the invasive phenotype of *Shigella*. We will address also the involvement of VirF in mechanisms aimed at withstanding adverse conditions inside the host, indicating that this protein is emerging as a global regulator whose action is not limited to virulence systems. Finally, we will discuss recent observations conferring VirF the potential of a novel antibacterial target for shigellosis.

## Introduction

Bacterial pathogens must often survive within fundamentally diverse habitats. Dynamic adaptation to the surroundings depends on the ability to sense environmental variations and to respond in an appropriate manner. This involves drastic changes in the transcriptional program of the cell. The capability to swiftly modulate gene expression requires investment by the bacterium in numerous gene functions that not only allow adaptation to different milieus but also enable the cell to co-ordinately rework its response to mutable conditions (Cases and de Lorenzo, 2005). The complexity of gene expression in pathogenic bacteria can be viewed as an evolutionary response to the challenge of surviving in changing environments (McAdams et al., 2004). Many pathogenic *E. coli*, including *Shigella*, are able to live in complex environments and have evolved intricate control systems. The expression of their pathogenic phenotype is the result of a multifactorial process which requires the synthesis of a large set of virulence determinants that may not be simultaneously needed during all stages of the infection process. These determinants are controlled by global regulatory networks, integrating specific regulators with conserved housekeeping processes.

*Shigella* is a facultative intracellular pathogen causing human bacillary dysentery, also known as shigellosis, a highly infectious disease widespread in developing countries. Although usually self-limiting, shigellosis can be fatal, especially in children (Anderson et al., 2016; Njamkepo et al., 2016; The et al., 2016). The pathogenicity of *Shigella* relies on its capacity to kill macrophages and invade colonic epithelial cells. Bacteria multiply intracellularly and spread to adjacent cells, with consequent cell death and inflammatory destruction of the mucosa (Schroeder and Hilbi, 2008; Ashida et al., 2015). The invasive process requires the coordinated expression of several genes located on the chromosome as well as on a virulence plasmid (pINV) (Sansonetti et al., 1982; Parsot, 2005). The acquisition of the pINV, a large F-type plasmid, by horizontal gene transfer (HTG) constitutes one of the most critical events in the evolution of the pathogenic lifestyle of *Shigella* since it encodes crucial elements of the molecular machinery required for invasion and intracellular survival (Pupo et al., 2000; The et al., 2016). pINV plasmids isolated from different *Shigella* spp. share significant homology and carry about 100 genes and an equivalent number of IS sequences (Buchrieser et al., 2000; Yang et al., 2005). Most genes required for host cell invasion and macrophage killing are contained in a conserved 31 kb region which is arranged as a PAI-like structure (Maurelli et al., 1985; Sasakawa et al., 1988). This so-called “entry region” consists of 34 genes organized into two large, divergently transcribed clusters. It contains the genes encoding the Ipa proteins, their chaperons and a specific T3SS system (Parsot, 2005). Besides these structural genes, the entry region hosts two regulatory genes coding for VirB and MxiE, the transcriptional activators required for the sequential expression of most pINV virulence genes (Beloin et al., 2002; Mavris et al., 2002). Scattered along the pINV, outside the entry region, are other genes encoding proteins crucial for the invasive process: the IcsA protein, responsible for the recruitment and polymerization of the host actin at one pole of the bacterial cell (Bernardini et al., 1989); the PhoN2 protein, required for proper IcsA localization (Scribano et al., 2014); the OspG protein, involved in the modulation of the host innate immune response (Kim et al., 2005); the IpaH proteins, which interfere with the host ubiquitination-dependent protein degradation (Ashida and Sasakawa, 2015); and the VirF protein, the primary virulence regulator (Sakai et al., 1988; Prosseda et al., 1998).

In this review we will focus on VirF summarizing its structure, its sophisticated regulation, its role as major player in the cascade of events leading to the activation of the virulence program of *Shigella*, and its involvement in mechanisms aimed at withstanding adverse conditions inside the host. We will also address how these features confer VirF the potential of a novel antibacterial target for shigellosis.

## VirF, an AraC-like regulator

VirF is a member of the AraC family of transcriptional regulators. This large family contains more than 800 different proteins, found mainly among gram negative bacteria and involved in the regulation of carbon metabolism, stress response, and virulence (Gallegos et al., 1997; Egan, 2002). The AraC proteins are characterized by two structural domains: a conserved C-terminal DNA binding domain and a more variable N-terminal signaling domain. The two domains are connected by an unstructured linker. DNA binding involves two helix-turn-helix (HTH) motifs and has been studied by analyzing the structure of the MarA (Rhee et al., 1998) and Rob (Kwon et al., 2000) proteins in complex with their target DNA, leading to the proposal that two different modes of DNA binding might exist involving either one (Rob) or both (MarA) HTH motifs. The N-terminal region is responsible for multimerization and/or binding of cofactors (Egan, 2002). The AraC family has been traditionally divided into three classes (Gallegos et al., 1997). The first one consists of proteins which, like AraC, act as regulators in response to a chemical signal (Schleif, 2010). Proteins involved in stress response, such as MarA and Rob, constitute the second class. VirF belongs to the third class, whose members control transcription in response to a physical signal (like temperature) and mostly serve as virulence gene regulators. While the proteins of the first and third class act as homodimers, proteins of the second class operate mainly as monomers. Members of the AraC family are frequently insoluble proteins. Precise molecular characterization of VirF and detailed information on its mechanism of action are still scarce as purification of VirF has been obtained only in a few cases (Porter et al., 2004; Tran et al., 2011). Most groups (Tobe et al., 1993; Porter and Dorman, 2002; Koppolu et al., 2013; Emanuele et al., 2014; Emanuele and Garcia, 2015) have reported difficulties in isolating VirF in quantities suitable to *in vitro* analysis and have therefore used a MalE—VirF fusion protein which still retains VirF functionality despite the lack of the first 10 N-terminal aminoacids (Tobe et al., 1993; Koppolu et al., 2013; Emanuele and Garcia, 2015).

VirF carries the two canonical AraC DNA-binding HTH motifs within its C-terminus. To acquire structural and functional information about the VirF protein, the entire *virF* gene has been subjected to both, random and site-directed mutagenesis and mutated proteins have been assayed for their ability to activate the plasmid-encoded invasive genes by analyzing the expression of a *mxiC-lacZ* fusion (Porter and Dorman, 2002). Mutating the key residues of the first HTH motif, in particular in the positioning helix (I180) or in the recognition helix (K193), inactivates VirF. The second HTH motif is essential as well, since mutating the key residues (Y239 and I241), which according to the MarA-DNA co-crystal (Rhee et al., 1998) form specific contacts with DNA, leads again to VirF inactivation. The functionality of VirF is hampered also by modifications in the hydrophobic core of HTH2 or by deletion of the C-terminal HTH2 region. This strongly suggests that VirF interacts with its target sequences via both HTH motifs. Additional evidence on the relevance of the two motifs to DNA binding stems from studies on another AraC regulator, PerA, which shares significant homology with VirF. This protein is required for the activation of the bundle-forming pili in ETEC and is able to fully substitute VirF in the activation of the *Shigella virB* promoter (Porter et al., 2004). Mutations affecting the DNA-interacting nucleotides from either of the two C-terminal HTH motifs of PerA have been shown to inactivate the protein, indicating that also PerA requires both HTH motifs to interact with its DNA targets (Porter et al., 2004). However, while PerA and Rns, another AraC-like regulator from ETEC (Porter et al., 1998), are able to complement *Shigella virF* mutants, VirF is unable to restore the expression of the PerA or Rns regulated genes, suggesting that a particular DNA structure which forms only at *Shigella* VirF-regulated promoters is required as signal for the activation of the VirF protein (Porter and Dorman, 2002; Porter et al., 2004).

AraC and several members of the AraC family are known to form homodimers (Gallegos et al., 1997; Egan, 2002; Schleif, 2010) and the residues required for self-association are contained in the N-terminal domain. As yet the only evidence suggesting that VirF may act as dimer/oligomer stems from the observation that two mutants unable to bind DNA are dominant negative when co-expressed with the wild type VirF. However, the fact that other HTH1 or HTH2 mutations have no trans-dominant effect rises the possibility (Porter and Dorman, 2002) that VirF binds first as monomer and then, following DNA bending and DNA-DNA interactions, forms a multisubunit nucleoprotein complex, as previously observed in the case of the melibiose-dependent activator MelR (Bourgerie et al., 1997). This is consistent with the observation that a MalE—VirF fusion protein recognizes a single, large (about 100 bp) region on the *virB* promoter (Tobe et al., 1993). However, recent footprinting analyses using purified VirF and revealing the presence of four distinct VirF binding sites (each spanning 40–60 bp) within the *icsA-RnaG* regulatory region (Tran et al., 2011) indicate that, rather than interacting at a single large spot, VirF recognizes its target sites with different affinities and may give rise to a large nucleoprotein complex only at higher protein concentration.

## VirF is at the top of the virulence regulatory cascade

**Figure 1:**
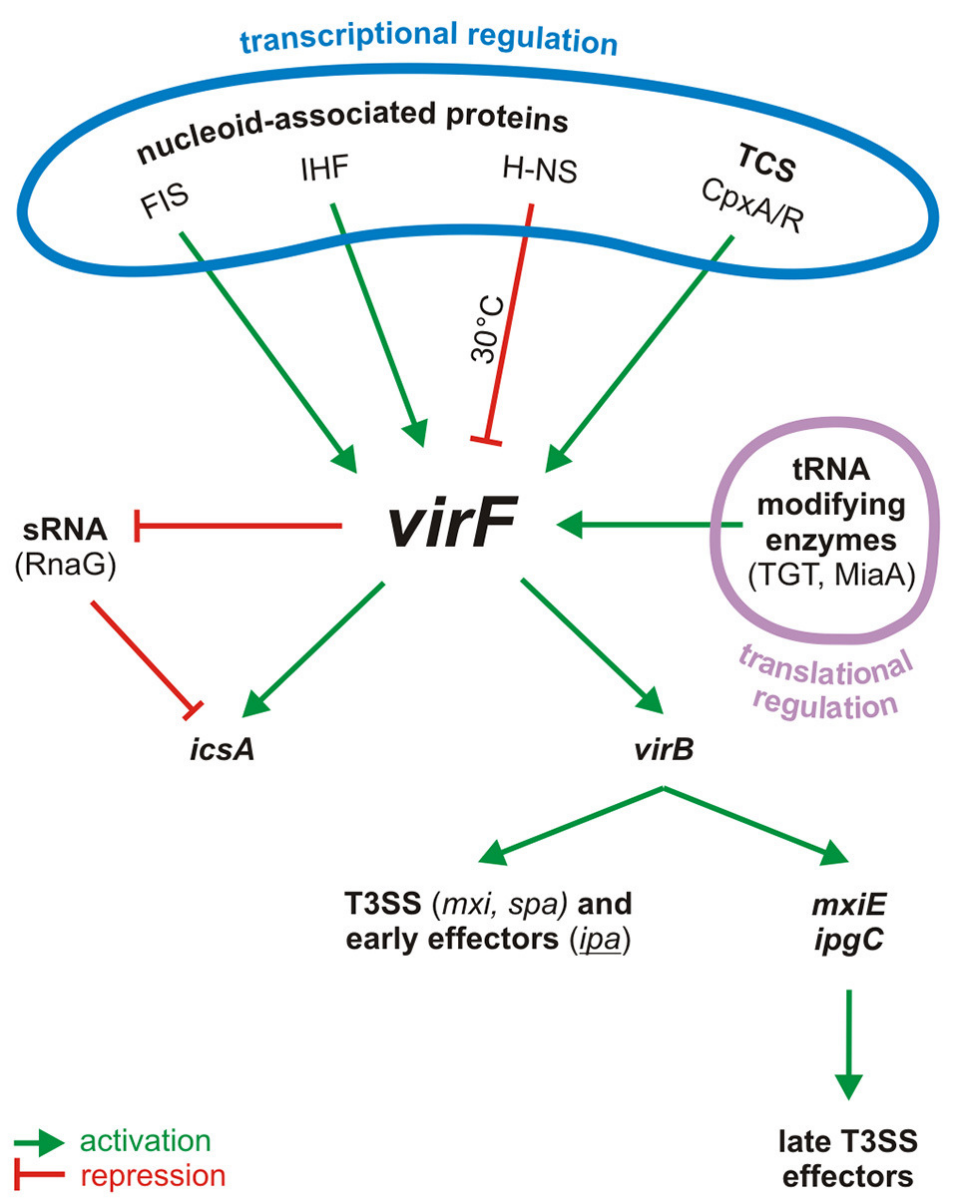
**Centrality of VirF in the pINV regulatory cascade of ***Shigella*****. The control of VirF occurs at transcriptional and translation levels. The major proteins involved in the regulation of VirF in response to environmental stimuli and nutrient conditions are indicated. Once synthesized, VirF activates the *icsA* and *virB* genes and represses the synthesis of the sRNA RnaG. VirB, the second regulator, then activates several operons, including those for a T3SS system and for the early effectors. VirB activates also the last regulator, MxiE, which in association with IpgC activates the late effectors. TCS, two component system.

The transcriptional activation of the invasive operons relies on the response to environmental stimuli like temperature, pH, and osmolarity, commonly encountered in the human intestine (Schroeder and Hilbi, 2008). This process requires the VirF protein (Sakai et al., 1988; Adler et al., 1989; Falconi et al., 1998; Durand et al., 2000) which, through a sophisticated regulatory cascade (Dorman and Porter, 1998; Prosseda et al., 2002; Parsot, 2005), leads to the full expression of the virulence phenotype (Figure 1). Temperature is a crucial factor since transcription of the pINV invasion genes is strongly repressed at 30°C by the chromosome-encoded protein H-NS (Maurelli and Sansonetti, 1988; Falconi et al., 1998). The primary event following the upshift of *Shigella* to the host temperature (37°C) is the synthesis of VirF which acts as an antisilencer, relieving H-NS mediated repression at the *virB* (Tobe et al., 1993) and *icsA* (Tran et al., 2011) loci. This is not uncommon: in many bacterial pathogens H-NS, one of the major components of the bacterial nucleoid, acts as transcriptional silencer of virulence genes located on mobile genetic elements (Dorman, 2004, 2007). As for the *icsA* gene, it encodes an outer membrane protein which promotes the intra- and intercellular spreading of *Shigella* among the epithelial cells of the colon (Bernardini et al., 1989; Lett et al., 1989). The other VirF-activated gene encodes a secondary transcriptional activator, VirB, which antagonizes the repressive activity of H-NS at the promoters of several pINV virulence genes by remodeling the DNA within H-NS-DNA nucleoprotein complexes (Beloin et al., 2002; McKenna et al., 2003). In particular, VirB activates, among others, the genes coding for the *Shigella* T3SS (Mxi and Spa proteins), for its early effectors and their chaperones (Ipa and Ipg proteins), and for MxiE, the last regulator in the cascade (Parsot, 2005; Schroeder and Hilbi, 2008). MxiE is another AraC-like protein (Kane et al., 2002; Mavris et al., 2002) which positively controls the expression of the late effectors but it becomes available as activator only when the IpgC chaperone facilitates its release from an anti-activator complex (OspD/Spa15).

The role of VirF as positive activator was first evidenced by observing that in *S. flexneri* (Sakai et al., 1988) and in *S. sonnei* (Kato et al., 1989) the expression of the four Ipa antigen proteins and of IcsA (known also as VirG) is silenced in the absence of VirF and increased when VirF is over expressed. It was then demonstrated (Adler et al., 1989; Tobe et al., 1991) that the regulatory activity of VirF is exerted directly when it interacts with the *icsA* gene, while it occurs indirectly, i.e., through the mediation of VirB, on the *ipa* operons. This gave the first basis for the classic cascade model. Originally, the regulatory role of VirF remained unclear and the protein was simply regarded as the element responsible for the capacity of *Shigella* to bind Congo red, a phenotype (Pcr^+^) associated with the expression of virulence (Sakai et al., 1986a). Initial molecular analyses of the pINV had led to the identification of an about 1 kb region responsible for the Pcr^+^ phenotype in *E. coli*. This region turned out to be located within the F fragment of a *SalI* pINV restriction digests and contained a gene, hence named *virF*, which was shown to be essential but not sufficient for the full expression of virulence in *S. flexneri* (Sakai et al., 1986a,b). Additional support to the active role played by VirF as regulator banks on the observations that its intracellular concentration is strictly related to the expression of virulence genes, that a VirF threshold level is required for the activation of the second regulator, *virB*, and that overexpression of *virF* fully restores the invasive phenotype at non-permissive temperature or in pINV-integrated strains (Adler et al., 1989; Dagberg and Uhlin, 1992; Colonna et al., 1995; Prosseda et al., 1998; Durand et al., 2000).

Cloning and sequencing analyses of the pINV plasmid have revealed that, in contrast to *virB* which is localized within the 31 kb PAI, the *virF* gene constitutes a “desert island” located 60 kb outside the PAI and surrounded by a mosaic of IS sequences (Buchrieser et al., 2000; Venkatesan et al., 2001; Prosseda et al., 2006), suggesting that it might have been acquired by the plasmid genome independently from the “entry region” (Prosseda et al., 2002). Besides its position, one of the striking features of the *virF* sequence is its low GC content—about 30%; interestingly this is true also for other virulence-related genes in the 31 kb PAI—as compared to that of the whole pINV plasmid and of the *Shigella* chromosome, which are about 48 and 50%, respectively (Buchrieser et al., 2000; Venkatesan et al., 2001; Yang et al., 2005). The *virF* gene exhibits canonical −35 and −10 regulatory boxes, however no clear ribosome binding site is found. While attempting to purify the protein it has been observed that VirF exists in three different forms: 30, 27, and 21 kDa (Sakai et al., 1986b; Kato et al., 1989). The larger form has been considered the active one while as yet no functional activity has been attributed to the two minor forms.

Strong evidence that VirF acts as a DNA binding protein capable to activate *virB* transcription by interacting with the upstream region of the *virB* promoter comes from the studies of Tobe et al. (1993). By deletion analysis of the *virB* promoter the authors have shown that the activation of *virB* requires a region of 110 bp upstream the transcription start site. The relevance of this region has been confirmed in the same study by footprinting with a MalE—VirF fusion protein. This allowed to identify a VirF binding site spanning from −117 to −17 which, by *in vitro* transcription, proved to be essential for the activation of *virB*. Considering the position of the VirF binding site and its role as activator, it has been proposed that VirF could act either by recruiting RNA polymerase at the *virB* promoter or by improving the ability of RNA polymerase to form an open complex (Tobe et al., 1993). The positive effect of VirF on *virB* transcription is counteracted by H-NS which has been shown to occupy a *virB* promoter region (spanning from −20 to +20) encompassing the RNA polymerase binding site (Tobe et al., 1993). Since the H-NS and VirF binding sites at the *virB* promoter are contiguous, it has been speculated that the binding of VirF may disrupt the H-NS-DNA repression complex, possibly being favored by thermally-induced changes in local supercoiling (Tobe et al., 1993). The relevance of superhelicity *per se* is supported by the finding that *virB* activation by VirF occurs much more efficiently on supercoiled templates than on relaxed ones (Tobe et al., 1993).

As mentioned before, also the *icsA* gene is controlled by VirF (Sakai et al., 1988). This gene encodes a protein responsible for the motility of *Shigella* and is located on the pINV plasmid outside the “entry region” (Bernardini et al., 1989; Lett et al., 1989). In contrast to all other structural genes involved in the invasive process, *icsA* does not require VirB (Figure 1). As is the case of *virB*, also *icsA* is repressed by the binding of H-NS to its regulatory region (Prosseda et al., 1998). This occurs at three sites (H-NS I, H-NS II, and H-NS III) and severely reduces *icsA* transcription at 30°C (Tran et al., 2011). Recent evidence shows that the regulation of *icsA* also depends on RnaG, a small antisense RNA transcribed on the complementary strand of *icsA* (Giangrossi et al., 2010). RnaG downregulates *icsA* transcription by means of two independent mechanisms, transcriptional interference, and transcriptional attenuation. In the first case the expression of RnaG decreases the activity of the *icsA* promoter. In the second case RnaG causes a premature termination of the *icsA* transcript (Giangrossi et al., 2010).

**Figure 2:**
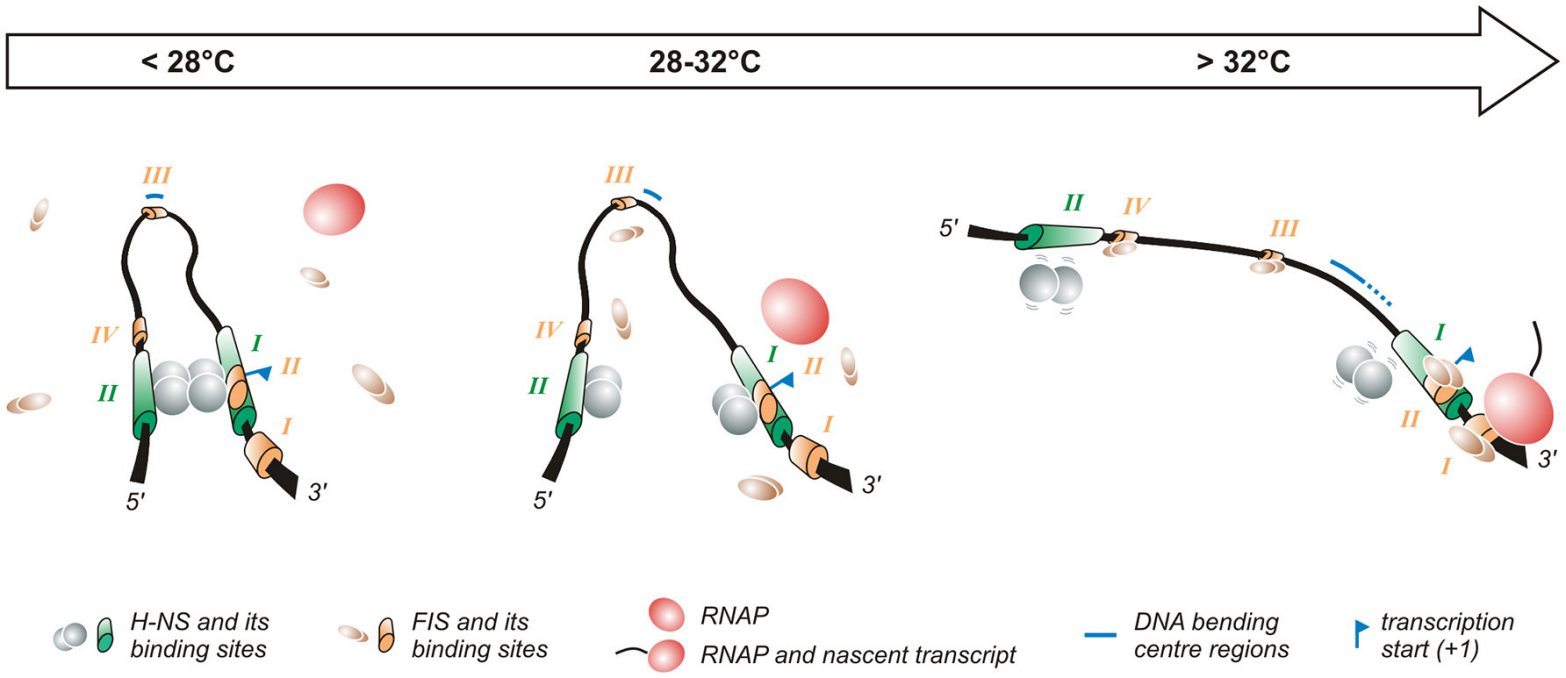
**Temperature-dependent regulation of the promoter of the ***virF*** gene**. The *virF* promoter contains two H-NS (indicated in green) and four FIS (indicated in orange) binding sites (Falconi et al., 1998, 2001). The arrow on the top of the figure indicates the transition from the outer environment (colder) to the intestinal host milieu (warmer). *Left panel*: At lower temperature a compact structure exists where H-NS represses *virF* by interacting with H-NS binding sites I and II which are separated by a DNA region whose sequence-mediated curvature is favored by the lower temperature. *Middle panel*: The intrinsic bend located between the two H-NS binding sites melts abruptly around 32°C and the bending center slides downstream unmasking a binding site for FIS. *Right panel*: By increasing the temperature the DNA bend relaxes further, H-NS interactions to binding sites I and II weaken and FIS gains easier access to its binding boxes, thus counteracting H-NS repression. Altogether these events lead to the formation of an active transcription complex.

Experiments performed with purified VirF indicate that this protein is able to directly stimulate the expression of *icsA* and that it binds to four 40–60 bp sites, two of which overlap the *icsA* and RnaG promoters (Tran et al., 2011). This region hosts H-NS sites I and II. The relative position of binding sites for H-NS and VirF provides a physical basis for a possible functional competition between the two proteins. By monitoring the *icsA* promoter activity in the presence of both H-NS and VirF it has been shown that VirF is able to significantly counteract the H-NS-dependent inhibition at the *icsA* promoter, thus acting as an anti-H-NS protein. VirF expression increases rapidly when temperature is raised above 32°C (Falconi et al., 1998; Prosseda et al., 1998; Durand et al., 2000; Figure 2). It is possible to speculate that at the host temperature (37°C) the increased amount of VirF may facilitate the interaction with its sites and consequently disrupt the H-NS-DNA complexes by forming a putative H-NS-*icsA*-VirF intermediate able to promote the switch from a repressed to an active state (Tran et al., 2011). Besides inducing *icsA* activation, VirF is also able to repress the expression of RnaG (Figure 1) by binding to a specific site overlapping the RnaG promoter. Thus, VirF promotes *icsA* expression in two ways: by acting on *icsA* both directly and indirectly, i.e., via repression of RnaG transcription (Tran et al., 2011). Altogether the experimental evidence existing on the role of VirF as major regulator in the cascade of events regulating the virulence of *Shigella* highlights the antagonism between H-NS and AraC proteins, suggesting that this may be a common evolutionary strategy that pathogens adopt to control virulence genes (Egan, 2002; Dorman, 2004).

## The regulation of the *virF* gene

The switch from the non-invasive to the invasive phenotype in *Shigella* is an elaborate process and the complexity of the regulatory mechanisms the *virF* gene undergoes is therefore not surprising. The expression of *virF* is affected by different environmental signals that act through highly diversified mechanisms. Nucleoid-associated proteins are known for their contribution to the transcriptional control of several genes including virulence genes (Rimsky and Travers, 2011). This is well exemplified in the case of *virF* (Prosseda et al., 2002) where H-NS (Falconi et al., 1998), FIS (Falconi et al., 2001), and IHF (Porter and Dorman, 1997b) are deeply involved as regulative elements.

The first studies aimed at understanding the temperature-regulated expression of the invasion phenotype of *Shigella* demonstrate that H-NS, originally designated *virR* (Maurelli and Sansonetti, 1988; Hromockyj et al., 1992), is responsible for silencing invasive genes at 30°C. Indeed, *hns*-defective mutants were shown to express the virulence determinants also at non-permissive temperature. Successive reports have revealed that the thermo-dependent expression of *virF* is lost in a *Shigella hns*-defective background, providing clear evidence that H-NS is able to affect the regulation of the *virF* gene (Dagberg and Uhlin, 1992; Colonna et al., 1995) and that this capacity is based on the ability of H-NS to bind the *virF* promoter with strong specificity (Prosseda et al., 1998). A detailed analysis (Falconi et al., 1998) of how H-NS interacts with the *virF* gene indicates that, both *in vivo* and *in vitro*, this protein is able to recognize and repress *virF* only below a critical temperature (32°C). This temperature-dependence relies on the interaction of H-NS with two binding sites within the *virF* promoter, spaced by a DNA linker region. The accessibility of the target DNA to H-NS varies significantly with temperature and H-NS is able to recognize its binding boxes only at lower temperature: with increasing temperature H-NS binding decreases, and at a temperature higher than 30°C the *virF* promoter becomes insensitive to H-NS repression. The position of the two H-NS binding sites on the *virF* promoter has been investigated in detail (Falconi et al., 1998). They have been mapped through *in vivo* and *in vitro* footprinting experiments and are localized around positions −250 (binding site II) and −1 (binding site I) with respect to the *virF* transcription start site. Site I overlaps the canonical −35 and −10 regulatory elements, suggesting that this region is involved in the transcriptional repression of the *virF* gene by H-NS: the interaction of H-NS with DNA may prevent the −35 and −10 boxes to be accessed by RNA polymerase (Figure 2). Binding site II maps far upstream the −35 and −10 sequences. In the absence of this site, H-NS forms a very unstable nucleoprotein complex, which cannot compete with RNA polymerase effectively. The DNA linker region separating the two H-NS binding sites is endowed with sequence-mediated curvature, a feature whose amplitude is inversely dependent on temperature (Prosseda et al., 2004, 2010). This DNA bend progressively relaxes with increasing temperature and is rapidly abolished above 32°C (Figure 2).

The fact that H-NS binding to the *virF* promoter, bending of the promoter region and repression of *virF* transcription are all sharply decreased by raising the temperature above a critical threshold suggests that the physical basis for the thermoregulated expression of *virF* resides in a temperature-dependent structural transition of the *virF* promoter, with the curved DNA tract within the promoter operating essentially as a thermosensor (Falconi et al., 1998). According to this model, with increasing temperature the transcriptionally inactive DNA architecture prevailing at low temperature would be replaced by a more relaxed geometry which no longer hinders the formation of a functional transcription complex (Falconi et al., 1998). This view has received further support by successive experiments (Prosseda et al., 2004) where the decreased DNA curvature of the *virF* promoter induced by raising the temperature is mimicked, at constant temperature, by templates (obtained by a targeted *in vitro* mutagenesis procedure) endowed with decreasing intrinsic DNA curvature. The results of footprinting and *in vivo* transcription assays using these templates highlight a strict correlation between intrinsic DNA curvature and the capability of H-NS to bind to its target sites and repress *virF* transcription. Moreover, the reciprocal rotational position of the two H-NS sites appears crucial for the thermoregulation of *virF*: moving the sites by about a half helix turn further apart produces templates which, despite a comparable overall bending level, are almost insensitive to H-NS repression, likely because the protein can no longer form a tight, transcription-blocking nucleoprotein complex. Temperature does not only affect the amplitude of the DNA bend within the *virF* promoter but also the position of the bending center, identified by means of circular permutation assays (Prosseda et al., 2004): at 4°C this spot maps at position −137 but with increasing temperature it slides downstream (up to position −55 at 60°C) within the region bounded by the two H-NS sites (Figure 2).

The complexity of the interaction between H-NS and bent DNA is increased further by the participation of FIS (Falconi et al., 2001). In agreement with the well-known growth phase dependent expression of FIS (Rimsky and Travers, 2011), the effect of this protein is higher in early exponential phase (Falconi et al., 2001), suggesting that it contributes to rapidly increase *virF* expression once bacteria have entered the host environment. FIS exerts, both *in vivo* and *in vitro*, a direct positive control on the transcription of *virF* and is able to bind to the *virF* promoter. Four FIS binding sites (I to IV), centered around positions +55 (site I), −1 (site II), −130 (site III, almost coinciding with the −137 position of the DNA bending center), and −200 (site IV), have been identified in the *virF* promoter region (Falconi et al., 2001). The interaction of FIS with site II likely hampers H-NS binding to its boxes. The downstream sliding of the DNA bending center and the concomitant curvature decrease occurring with increasing temperature favor the binding of FIS to the other sites inducing an adjustment to the geometry of the DNA that hinders long range H-NS/H-NS interaction, thus promoting the transcription of *virF*. Several reports have stressed the validity of this model—envisaging an environmentally induced structural collapse of a promoter's intrinsic bend as a regulative key—also among other pathogenic bacteria, like *Y. enterocolitica virF* (Rohde et al., 1999), and *E. coli hly* (Madrid et al., 2002).

Besides FIS also IHF is involved in the positive control of the pINV regulon by stimulating the transcription of the *virF* and *virB* genes (Porter and Dorman, 1997b). IHF is composed by two subunits encoded by the *ihfA* and *ihfB* genes (Rimsky and Travers, 2011). IHF binds to the *virF, virB*, and *icsA* promoters. In the case of *virF* it recognizes a 127 bp fragment spanning from the promoter-proximal sequence to the start of the ORF, with a putative binding site located between +45 and +57. In *ihfA*-defective strains entering stationary phase the expression of the Ipa proteins and of the T3SS components is decreased as a consequence of the reduced level of VirF and VirB (Porter and Dorman, 1997b). This growth-phase-dependent effect is consistent with the increased level of IHF during late growth phases (Rimsky and Travers, 2011). However, the observation that in a *ihfA* mutant virulence gene expression is reduced only two- to three-fold suggests that IHF, rather than constituting an absolute requirement, plays a modulatory role.

The expression of the *Shigella* invasive genes is repressed at low pH and low osmolarity (Porter and Dorman, 1997a). Also in these cases regulation occurs mainly at the level of *virF* expression. While it has been demonstrated that H-NS is involved, at least in part, in the repression of *virF* under low osmotic conditions (Mitobe et al., 2009), the pH-dependent regulation of *virF* requires the two component regulatory system CpxA/CpxR (Nakayama and Watanabe, 1995, 1998; Figure 1). The Cpx system responds to a broad range of stimuli including, besides pH, also salt, metals, lipids, and misfolded proteins that cause perturbations in the bacterial envelope (Raivio, 2014). In particular, it has been shown that the response regulator CpxR binds directly to a fragment containing the upstream promoter region of *virF* between positions −103 and −37. Phosphorylation of CpxR enhances its binding capacity and directly activates the transcription of *virF* (Nakayama and Watanabe, 1998). Due to the relevance of the CpxA/CpxR system in response to several stimuli connected to envelope stress, the integration of *virF* within the CpxR-controlled network represents a further regulatory layer acting on the fine control of VirF.

Besides being subject to a complex transcriptional control the *virF* gene is also regulated at the translational level. It has been shown that post-transcriptional modifications of tRNA affect the translation of VirF (Figure 1). The full expression of *S. flexneri* virulence genes depends on the presence of two modified tRNA nucleosides (queuosine, at position 34, and 2-methylthio-N6-isopentenyladenosine, adjacent to 3′-end of the anticodon). The synthesis of these nucleosides depends on the products of the *tgt* (encoding TGT, the tRNA-guanine transglycosylase) and *miaA* (coding for MiaA, a tRNA-N^6^-isopentyladenosine synthetase) genes, respectively. The intracellular concentration of VirF decreases in *tgt* mutants and in *miaA* mutants, inducing a low virulence phenotype and an avirulent phenotype, respectively (Durand et al., 1994, 1997). Overall, tRNA modifications are required for VirF to reach the threshold level necessary to activate the virulence cascade (Durand et al., 2000). Besides being involved in tRNA modification TGT also recognizes (Hurt et al., 2007) the *virF* mRNA *in vitro* and this recognition results in a site-specific modification at position 421 responsible for the change from guanine to adenine in the mRNA. As yet the physiological significance of this modification is unclear. A contribution of tRNA modification enzymes in virulence has been reported also in other systems: the GidA and MnmE enzymes, which act together as a complex, are both required for the expression of the full virulence phenotype in *Salmonella enterica, Aeromonas hydrophila*, and in the plant pathogen *Pseudomonas syringae* (Shippy and Fadle, 2014). Morevoer, the GidA/MnmE complex has been shown to be relevant for the stress response in *Salmonella* and MiaA is essential in *E. coli* for growth at higher temperature (Tsui et al., 1996).

Finally, a connection between bacterial metabolism and virulence gene expression has been found in *S. flexneri*, observing that the addition of ornithine to minimal medium reduces *virF* expression while the addition of putrescine, lysine and few other aminated metabolites counteract this inhibition (Durand and Björk, 2003, 2009). Moreover, it has been observed that proteins involved in the glycolytic pathway, most notably the carbon regulator CsrA, are required for the full expression of the *Shigella* virulence phenotype: indeed, the inactivation of *csrA* causes a significant reduction of *virF* and *virB* expression (Gore and Payne, 2010). As yet the molecular mechanism adopted by CsrA to control these genes is unknown.

## VirF as global regulator

The evolution of bacterial pathogens from harmless ancestors mainly depends on the acquisition of virulence gene clusters on plasmids, phages, and pathogenicity islands by HGT. This process is complemented by the progressive adaptation to a specific niche determined by so-called pathoadaptive events such as mutations, rearrangements or deletions of genes unnecessary, or even deleterious, for optimal fitness to the new environment (Ochman and Moran, 2001). These events usually involve the concomitant acquisition or loss of regulatory factors which modify the transcriptional profile of the host to a significant extent. The evolution of *Shigella* from *E. coli* is a most studied exemplification of these events (Prosseda et al., 2012).

Global transcriptional analyses of *E. coli* cells expressing or lacking the *virF* gene have contributed to understand to what extent the arrival of VirF by the acquisition of the pINV plasmid in *Shigella* has altered the transcriptional program of the ancestor. These studies have revealed that the activity of VirF is not restricted to the regulation of plasmid-encoded virulence genes as *icsA* and *virB* but extends also to chromosomally-located genes (Barbagallo et al., 2011). Genes activated by VirF can be grouped into two categories: those which are functional both in *Shigella* and *E. coli*, and those that have been inactivated in *Shigella*. Among the more VirF-sensitive genes of the first group there are those encoding the heat shock proteins IbpA, GroESL, HtpG, DnaK, and Lon. Given the protective role exerted by these stress proteins, it can be hypothesized that the functional significance of VirF resides in activating genes which contribute to better withstand adverse conditions inside the host.

The existence of VirF-regulated genes silent in *Shigella* (i.e., the second group) suggests that some of them may encode factors perturbing the invasive process which likely have been silenced during evolution in order to optimize bacterial fitness within the host. Most of these genes encode yet unknown products. In this respect the *speG* gene, encoding the enzyme spermidine acetyl transferase (SAT), is an exception. In *E. coli*, SAT prevents the accumulation of spermidine (and the consequent toxic effects) by catalyzing the conversion of this polyamine into its physiologically inert form, acetylspermidine (Barbagallo et al., 2011). The *speG* gene belongs to the *ynfB*-*speG* operon (no function is as yet known for the *ynfB* product), whose induction by VirF has been observed only at 37°C (Barbagallo et al., 2011) in agreement with the thermodependency of *virF* expression (Falconi et al., 1998). The loss of *speG* constitutes a pathoadaptive mutation (Prosseda et al., 2012; Campilongo et al., 2014) since spermidine accumulation induced by the lack of SpeG activity increases *Shigella* survival inside macrophages and its resistance to oxidative stress conditions (Barbagallo et al., 2011). These data have led to hypothesize that, during the evolutionary transition from *E. coli* to *Shigella*, the acquisition of *virF* on the pINV plasmid might have caused an increased expression of *speG*, thus lowering the intracellular spermidine content (Di Martino et al., 2013). Evidence supporting this view stems from the observation that in *Shigella speG* expression is decreased in a VirF-defective background and that restoration of *speG* expression lowers the bacterial fitness within macrophages (Barbagallo et al., 2011).

Recently VirF has been shown to be involved in the regulation of *mdtJI*, another operon related to polyamine function in bacteria. In *E. coli* the *mdtJI* operon, which encodes an efflux pump belonging to the small multidrug resistance family of transporters, is almost silent because of a strong repression by H-NS (Leuzzi et al., 2015). Despite the high homology between *Shigella* and *E. coli*, in *Shigella* the *mdtJI* pump is expressed because the H-NS repressive effect is counteracted by VirF and favored by high levels of spermidine (Leuzzi et al., 2015). Intracellular spermidine does not affect the synthesis of VirF (Leuzzi et al., 2015) and the molecular mechanism by which this polyamine contributes towards the activation of *mdtJI* is still unclear. Genetic studies on the expression of *mdtJI* in *Shigella* indicate the presence of a VirF binding site around the promoter consensus boxes which partially overlaps an H-NS site, supporting the occurrence of a competition between the two proteins (Leuzzi et al., 2015), in close analogy with the observations on the *virB* and *icsA* promoters (Tobe et al., 1993; Tran et al., 2011). In *Shigella* MdtJI promotes the secretion of putrescine, the precursor of spermidine, and it has been proposed that VirF- and spermidine-mediated activation of the *mdtJI* operon represents a safety mechanism allowing spermidine to accumulate within the bacterial cell to a level optimally suited for survival within infected macrophages and, at the same time, avoid toxic side effects on bacterial viability due to spermidine excess (Leuzzi et al., 2015). Altogether, the observations on the ability of *Shigella* to activate chromosomal genes evidence the extent to which the acquisition of a new regulator by HGT represents a crucial event for reshaping the transcriptional profile of the core genome, facilitating bacterial adaptation to specific niches within infected hosts.

## VirF as novel target for anti-virulence therapies

Each year *Shigella* is responsible for 125 million cases of illness, mainly in low income countries (The et al., 2016). Despite the enormous clinical relevance of these infections and the emergence of multiresistance strains, no vaccine has been as yet released for public use (Anderson et al., 2016). Several recent studies have focused on the development of novel treatment strategies targeting virulence instead of bacterial viability, since this is regarded as a highly effective approach to combat bacterial infections while minimizing the emergence of antibiotic resistances (Rasko and Sperandio, 2010). The expression of virulence factors is not required for cell viability and therefore bacterial pathogens should be subject to less selective pressure to develop resistance to inhibitors of virulence determinants. AraC proteins are considered very interesting candidate targets in anti-virulence strategies due to their critical role in controlling virulence in pathogenic bacteria (Rasko and Sperandio, 2010). Specific inhibitors can affect AraC-mediated processes at different stages, such as self-association, DNA binding, and recruitment of RNA polymerase.

As for shigellosis it is therefore not surprising that VirF has been considered a very good antivirulence target, since its silencing prevents host cell invasion and intercellular spreading without affecting bacterial viability (Sakai et al., 1988; Falconi et al., 1998). So far, two approaches have been adopted to identify potential VirF inhibitors: a high-throughput screening for small molecules and a targeted search among already characterized AraC inhibitors. In the first case (Hurt et al., 2010), the expression of *virB* in response to VirF has been analyzed using a pINV-cured *S. flexneri* strain harboring a recombinant plasmid containing the *virF* gene and a *virB-lacZ* fusion. The screening, performed initially on 42,000 compounds from several small-molecule libraries and then extended to an additional set of 100,000 compounds, has led to the identification of about 600 molecules meeting the selection criteria for VirF inhibition (Hurt et al., 2010; Emanuele et al., 2014). After selecting for candidates with favorable medicinal chemistry, low toxicity and a dose-dependent activity, five compounds able to inhibit VirF-driven transcriptional activation with very low IC_50_ values were considered for further studies. These compounds were characterized by the presence of aromatic or heterocyclic rings that could interact with DNA or with aromatic acids that typically associate with DNA (Hurt et al., 2010; Emanuele et al., 2014). *In vivo* assays on cell cultures have shown that only one polycyclic compound, named 19615 (methyl-[2-(2-phenyl-4a,9b-dihydro-benzo[4,5]furo[3,2-*d*]pyrimidin-4-γ1oxy9-ethyl]-amine), was able to severely affect intercellular spreading and induce a significant inhibition of cell invasion (Emanuele and Garcia, 2015). In particular this molecule has been demonstrated to be able to directly interact with a MalE—VirF fusion protein and inhibit its binding to the *virB* promoter. Further studies to structurally optimize the selected compound are required to fully clarify its effectiveness in an antivirulence therapy.

The second approach (Koppolu et al., 2013) adopted so far to identify possible VirF inhibitors has been centered on a small molecule, SE-1, a quinoline derivative [1-butyl-4-nitromethyl1-3-(quinolin-γl)-4Hquinoline] previously identified as an inhibitor of two AraC activators, RhaS, and RhaR (Skredenske et al., 2013). Since SE-1 interacts with the conserved DNA-binding domains of the AraC proteins (Skredenske et al., 2013), it has been considered able to potentially inhibit also other AraC activators. This turned out to be true: the data revealed that SE-1 induced a significant reduction in the expression of all VirF-controlled genes, consequently inhibiting the invasion of epithelial cells. SE-1 binds directly to a MalE—VirF fusion protein, a feature likely responsible for inhibiting VirF to interact with DNA and activate transcription. On account that SE-1 does not affect the growth of *Shigella* and does not have detectable toxicity in human cell cultures, it has been considered as another good candidate as novel antibacterial agent.

## Conclusions and perspectives

Altogether the available data stress how *Shigella*, in its long route to becoming a successful pathogen, has evolved an elaborate regulatory system to ensure the coordinated activation of virulence determinants or prevent their wasteful expression, depending on environmental signals. The complexity of the circuitry regulating virulence in *Shigella* highlights the relevance of the firing of a major regulator, the *virF* gene, to the successful development of the invasive program. Through studies analyzing the interplay, either synergistic or antagonistic, among nucleoid-associated proteins, sRNA, global, and specific regulators, and intrinsic features of the DNA, the complex nature of the regulation of VirF and of the genes under its control is emerging with intriguing detail. However, while the master regulator has been identified almost three decades ago, several open questions still exist, such as the capacity of VirF to form dimers or more complex aggregates and the molecular mechanisms underlying its DNA binding specificity and its interactions with RNA polymerase. Understanding how the gene regulatory circuitry has evolved in bacterial pathogens represents a challenge. From the evolutionary standpoint this relates to the need to understand how genes acquired by HGT have integrated into existing regulatory networks and how newly acquired regulators have shaped the genome of the new bacterial host. In the long run a better understanding of the structure of the VirF protein can be expected to positively impact on the development of new therapeutic approaches based on the use of specific inhibitory compounds.

## Author contributions

All authors listed, have made substantial, direct and intellectual contribution to the work, and approved it for publication. In particular MLDM, BC and GP conceived the review and wrote the manuscript. MLDM and GM conceived the figures. MF and GM critically edited the final version of the review.

## Funding

This research was supported by grants from Ministero della Ricerca e dell'Istruzione (PRIN 2012/WWJSX8K), Sapienza Università di Roma, Consiglio Nazionale delle Ricerche, and Institut Pasteur (PTR-24-16).

### Conflict of interest statement

The authors declare that the research was conducted in the absence of any commercial or financial relationships that could be construed as a potential conflict of interest.

## Abbreviations

FIS: factor for inversion stimulation
HGT: horizontal gene transfer
H-NS: heat stable nucleoid-structuring protein
HTH: helix-turn-helix
IC_50_: half maximal inhibitory concentration
IHF: integration host factor
Ipa: invasion plasmid antigen
IS: insertion sequence
MiaA: tRNA-N^6^-isopentyladenosine synthetase
PAI: pathogenicity island
PCr: Congo red phenotype
sRNA: small RNA
SAT: spermidine acetyl transferase
T3SS: type III secretion system
TCS: two component system
TGT: tRNA-guanine transglycosylase.
